# Effective cross-over to granisetron after failure to ondansetron, a randomized double blind study in patients failing ondansetron plus dexamethasone during the first 24 hours following highly emetogenic chemotherapy

**DOI:** 10.1054/bjoc.2001.2045

**Published:** 2001-10

**Authors:** R de Wit, A C de Boer, G H M vd Linden, G Stoter, A Sparreboom, J Verweij

**Affiliations:** Rotterdam Cancer Institute and University Hospital Rotterdam, IJsselland Hospital Rotterdam, Albert Schweitzer Hospital Dordrecht, The Netherlands

## Abstract

In view of the similarity in chemical structure of the available 5HT_3_-receptor antagonists it is assumed, whilst these agents all act at the same receptor, that failure to one agent would predict subsequent failure to all 5HT_3_-receptor antagonists. We conducted a randomized double blind trial of granisetron 3 mg plus dexamethasone 10 mg versus continued treatment with ondansetron 8 mg plus dexamethasone 10 mg in patients with protection failure on ondansetron 8 mg plus dexamethasone 10 mg during the first 24 hours following highly emetogenic chemotherapy. Of 40 eligible patients, 21 received ondansetron + dexamethasone and 19 received granisetron + dexamethasone. We found a significant benefit from crossing-over to granisetron after failure on ondansetron. Of the 19 patients who crossed over to granisetron, 9 patients obtained complete protection, whereas this was observed in 1 of the 21 patients continuing ondansetron, *P* = 0.005. These results indicate that there is no complete cross-resistance between 5HT_3_-receptor antagonists, and that patients who have acute protection failure on one 5HT_3_-receptor antagonist should be offered cross-over to another 5HT_3_-receptor antagonist. © 2001 Cancer Research Campaign  http://www.bjcancer.com


British Journal of Cancer (2001) 85(8), 1099–1101
© 2001 Cancer Research Campaign
doi: 10.1054/ bjoc.2001.2045, available online at http://www.idealibrary.com on IDEAL

### Effective cross-over to granisetron after failure to ondansetron, a randomized double blind study in patients failing ondansetron plus dexamethasone during the first 24 hours following highly emetogenic chemotherapy

**R de Wit, AC de Boer, GHM vd Linden, G Stoter, A Sparreboom and J Verweij**

Rotterdam Cancer Institute and University Hospital Rotterdam, IJsselland Hospital Rotterdam, Albert Schweitzer Hospital Dordrecht, The Netherlands

**Summary** In view of the similarity in chemical structure of the available $5HT_3$-receptor antagonists it is assumed, whilst these agents all act at the same receptor, that failure to one agent would predict subsequent failure to all $5HT_3$-receptor antagonists. We conducted a randomized double blind trial of granisetron 3 mg plus dexamethasone 10 mg versus continued treatment with ondansetron 8 mg plus dexamethasone 10 mg in patients with protection failure on ondansetron 8 mg plus dexamethasone 10 mg during the first 24 hours following highly emetogenic chemotherapy. Of 40 eligible patients, 21 received ondansetron + dexamethasone and 19 received granisetron + dexamethasone. We found a significant benefit from crossing-over to granisetron after failure on ondansetron. Of the 19 patients who crossed over to granisetron, 9 patients obtained complete protection, whereas this was observed in 1 of the 21 patients continuing ondansetron, $P$ = 0.005. These results indicate that there is no complete cross-resistance between $5HT_3$-receptor antagonists, and that patients who have acute protection failure on one $5HT_3$-receptor antagonist should be offered cross-over to another $5HT_3$-receptor antagonist.
© 2001 Cancer Research Campaign http://www.bjcancer.com

The introduction of $5HT_3$-receptor antagonists has meant a breakthrough in the protection against chemotherapy-induced acute emesis (Verweij et al, 1996). When combined with dexamethasone, $5HT_3$-receptor antagonists result in complete protection in 70–80% of patients receiving highly emetogenic chemotherapy (Gandara et al, 1998). The 2 most widely used agents are ondansetron (Zofran) and granisetron (Kytril). In Europe, the approved dose of ondansetron is 8 mg i.v., and of granisetron 3 mg i.v., both given as a single dose, prior to the administration of the chemotherapy (Kamanabrou, 1992; Gandara et al, 1998). Several trials comparing $5HT_3$-receptor antagonists have demonstrated equivalent anti-emetic efficacy (Ruff et al, 1994; Navari et al, 1995; Stewart et al, 1995; Perez et al, 1998). In view of the similarity in chemical structure these agents act at the same receptor, and it is assumed that failure to one $5HT_3$-receptor antagonists would predict subsequent failure to all $5HT_3$-receptor antagonists (Verweij et al, 1996; Gandara et al, 1998; Gralla, 1998). In a pilot experience we previously observed successful protection after crossing over between $5HT_3$-receptor antagonists (De Boer et al, 1995). In that uncontrolled pilot study several patients who had acute emesis protection failure on tropisetron were completely protected after cross-over to ondansetron. The present report involves a randomized double blind cross-over study of granisetron plus dexamethasone versus continued treatment with ondansetron plus dexamethasone, in patients with protection failure on ondansetron plus dexamethasone during the first 24 hours following highly emetogenic chemotherapy.

*Received 29 May 2001*
*Revised 13 July 2001*
*Accepted 16 July 2001*

*Correspondence to:* R de Wit

### PATIENTS AND METHODS

Eligibility required protection failure (defined as ≥ 2 vomits, severe nausea (no significant intake possible) or nausea > 4 hours) within 24 hours after single day cisplatin ≥ 50 mg $m^{-2}$ or cyclophosphamide ≥ 500 mg $m^{-2}$ based chemotherapy, on antiemetic prophylaxis with ondansetron 8 mg i.v. and dexamethasone 10 mg i.v. Eligibility also required that there were no planned dose attenuations, no use of other antiemetic agents, benzodiazepines, or opiates, and no emesis in the 24 hours preceding the study cycle.

After informed consent patients were randomized in a double blind fashion to granisetron 3 mg i.v. plus dexamethasone 10 mg i.v. or continued treatment with ondansetron 8 mg i.v. plus dexamethasone 10 mg i.v. The medication was prepared in blinded 50 ml saline bags by the pharmacist.

Results were documented by the patient on diary cards:

- Complete protection (CP) was defined as no vomiting and no or mild nausea.
- Partial protection (PP) was defined as 0–1 vomits, and/or moderate nausea during a maximum of 4 hours.
- Failure (F) was defined as ≥ 2 vomits, or severe nausea (no significant intake possible), or nausea lasting more than 4 hours.

### RESULTS

A total of 45 patients were randomized. 5 patients were excluded at the study cycle for the following reasons; nausea prior to the chemotherapy (2), chemotherapy dose reductions (2), other antiemetics (1). Of 40 eligible patients, 21 received ondansetron plus dexamethasone, and 19 crossed-over to granisetron plus dexamethasone. Patients were well balanced for age, sex, type and dose of chemotherapy and number of previous cycles (Table 1).

We found a significant benefit from crossing-over to granisetron after failure to ondansetron (Table 2). Of the 19 patients who crossed-over to granisetron, 9 patients obtained complete protection (CP), whereas this was observed in 1 of 21 patients continuing ondansetron, $P = 0.005$ (Fisher exact test). The $P$ value of a test for trend with ordered categories ($\chi^2$ trend) was $< 0.005$. Successful cross-over was observed both in patients receiving cisplatin, and in patients receiving cyclophosphamide-based chemotherapy, but the numbers in the subsets were too small to perform meaningful subanalyses.

### DISCUSSION

Several large well-designed randomized studies between ondansetron and granisetron have demonstrated equivalent antiemetic efficacy (Ruff et al, 1994; Navari et al, 1995; Stewart et al, 1995; Perez et al, 1998). Reviewing the more recently conducted randomized trials of a sample size that justifies to draw such conclusion, there appears no therapeutical difference between ondansetron doses ranging from 8 mg, given as a single dose before the start of the chemotherapy to 32 mg administered during the first 24 hours (Ruff et al, 1994; Italian Group of Antiemetic Research, 1995; Gandara et al, 1998). The same applies for granisetron single dosages of 1 and 3 mg (Navari et al, 1994, 1995; Morrow et al, 1995; Perez et al, 1997; Gralla, 1998; Martoni et al, 1998). In addition, there are no data to support the use of higher doses of the same $5HT_3$-receptor antagonists in patients failing the recommended dosage (Gandara et al, 1998).

Hence, it is unlikely that the high rate of successful complete protection by granisetron after failure to ondansetron in the present study can be explained by better dose-effectiveness of granisetron 3 mg as compared with ondansetron 8 mg. The results in our study indicate that there is no complete cross-resistance between these two $5HT_3$-receptor antagonists, at least not in the sequence ondansetron failure, followed by cross-over to granisetron. These findings lend support to our previous observation of successful cross-over between $5HT_3$ receptor antagonists, indicating that there is no complete cross-resistance between those agents (de Boer et al, 1995). In this pilot study, 5 of 14 patients who had emesis protection failure on tropisetron obtained complete protection after cross-over to ondansetron.

Therefore, we conclude that there is no cross-resistance between $5HT_3$-receptor antagonists and that patients who have acute protection failure on one $5HT_3$-receptor antagonist should be offered cross-over to another $5HT_3$-receptor antagonist.

**Table 1** Patient characteristics (eligible patients)

| | Granisetron ($n$ = 19) | Ondansetron ($n$ = 21) |
|---|---|---|
| Sex female/male | 18/1 | 18/3 |
| Median age (range) | 46 (29–71) | 46 (30–73) |
| Cisplatin-based chemotherapy | 7 | 6 |
| Cyclophosphamide-based chemotherapy | 12 | 15 |
| Previous cycles (number + range) | 2 (2–15) | 2 (2–13) |
| Tumour type | | |
| Breast | 11 | 14 |
| Ovarian | 3 | 1 |
| Lung | 2 | 2 |
| Other | 3 | 4 |

**Table 2** Results after cross-over

| Cisplatin-based chemotherapy | Complete protection | Partial protection | Failure |
|---|---|---|---|
| Ondansetron ($n$ = 6) | 0 | 2 | 4 |
| Granisetron ($n$ = 7) | 2 | 2 | 3 |
| **Cyclophosphamide-based chemotherapy** | Complete protection | Partial protection | Failure |
| Ondansetron ($n$ = 15) | 1 | 3 | 11 |
| Granisetron ($n$ = 12) | 7 | 2 | 3 |
| **Total** | Complete protection | Partial protection | Failure |
| Ondansetron ($n$ = 21) | 1 | 6 | 14 |
| Granisetron ($n$ = 19) | 9* | 3 | 7 |

*Fisher exact test (Complete Protection vs no Complete Protection), $P = 0.005$, $\chi^2$ test for trend, $P < 0.001$.

### REFERENCES

Boer de M, Wit de R, Stoter G and Verweij J (1995) Possible lack of full cross-resistance of $5HT_3$ antagonists; a pilot study. *J Cancer Res Clin Oncol* **121**: 126–127

Gandara DR, Roila F, Warr D, Edelman MJ, Perez EA and Grala RJ (1998) Consensus proposal for 5HT3 antagonists in the prevention of acute emesis related to highly emetogenic chemotherapy. Dose, schedule, and route of administration. *Support Care Cancer* **6**: 237–243

Gralla RJ (1998) Antiemetic therapy. *Sem Oncol* **25**: 577–583

Italian Group for Antiemetic Research (1995) Ondansetron versus granisetron, both combined with dexamethasone, in the prevention of cisplatin-induced emesis. *Ann Oncol* **6**: 805–810

Kamanabrou D (1992) Intravenous granisetron establishing the optimal dose. *Eur J Cancer* **28A**: S6–S11

Martoni A, Piana E, Strocchi E, Angelelli B, Guaraldi M, Zamagni C, Camaggi CM and Pannutti F (1998) Comparative crossover trial of two intravenous doses of granisetron (1 mg vs 3 mg) + dexamethasone in the prevention of acute cis-platinum-induced emesis. *Anticancer Res* **18**: 2799–2804

Morrow GR, Jane MS, Hickok JT and Rosenthal SN (1995) Progress in reducing nausea and emesis. Comparisons of ondansetron (Zofran), Granisetron (Kytril), and Tropisetron (Navoban). *Cancer* **76**: 343–357

Navari R, Gandara D, Hesketh P, Hall S, Maillard J, Ritter H, Friedman C and Fitts D (1995) Comparative clinical trial of granisetron and ondansetron in the prophylaxis of cisplatininduced emesis. *J Clin Oncol* **13**: 1242–1248

Navari RM, Kaplan HG, Gralla RJ, Grunberg SM, Palmer R and Fitts D (1994) Efficacy and safety of granisetron, a selective 5-hydroxytryptamine-3 receptor antagonist, in the prevention of nausea and vomiting induced by high-dose cisplatin. *J Clin Oncol* **12**: 2204–2210

Perez EA, Navari RM, Kaplan HG, Gralla RJ, Grunberg SM, Palmer RH and Fitts D (1997) Efficacy and safety of different doses of granisetron for the prophylaxis of cisplatin-induced emesis. *Support Care Cancer* **5**: 31–37

Perez EA, Hesketh P, Sandbach J et al (1998a) Comparison of single-dose oral granisetron versus intravenous ondansetron in the prevention of nausea and vomiting induced by moderately emetogenic chemotherapy: a multicenter, double-blind, randomized parallel study. *J Clin Oncol* **16**: 754–760

Perez EA, Lembersky B, Kaywin P, Kalman L, Yocom K and Friedman C (1998b) Comparable safety and antiemetic efficacy of a brief (30-second bolus)

 *© 2001 Cancer Research Campaign*

intravenous granisetron infusion and a standard (15-minute) intravenous ondansetron infusion in breast cancer patients receiving moderately emetogenic chemotherapy. *Cancer J Sci Am* **4**: 52–58

Ruff P, Paska W, Goedhals L, Pouillart P, Rivière A, Vorobiof D, Bloch B, Jones A, Martin C, Brunet R, Butcher M, Forster J and McQuade B (1994) Ondansetron compared with granisetron in the prophylaxis of cisplatin-induced acute emesis: a multicentre double-bline, randomized parallel-group study. *Oncology* **51**: 113–118

Stewart A, McQuade B, Cronje JDE et al (1995) Ondansetron compared with granisetron in the prophylaxis of cyclophosphamide-induced emesis in out-patients: a multicentre, double-blind, dboule-dummy, randomized, parallel-group study. *Oncology* **52**: 202–210

Verweij J, Wit de R and Mulder de PHM (1996) Optimal control of acute cisplatin-induced emesis. *Oncology* **53**: 56–64

© 2001 Cancer Research Campaign
